# Tympanosclerosis Presenting as Mass: Workup and Differential

**DOI:** 10.1155/2016/9821493

**Published:** 2016-08-30

**Authors:** Jonnae Y. Barry, Saranya Reghunathan, Abraham Jacob

**Affiliations:** Department of Otolaryngology-Head and Neck Surgery, The University of Arizona College of Medicine, Tucson, AZ, USA

## Abstract

*Introduction*. Tympanosclerosis is a commonly encountered entity within ENT clinics and primary care settings. Recognizing ear pathology is essential for correct management. Oftentimes the diagnosis is clear; however in certain cases further workup to rule out other more insidious disease processes is warranted.* Case Report*. We present a case of tympanosclerosis which presented as an ear mass without classic appearance of tympanosclerosis. Through imaging and biopsy the diagnosis of tympanosclerosis was made. The patient was treated surgically with good outcome.* Discussion*. Various ear pathologies, with different treatment algorithms, may present as clinically similar to one another. Differential diagnosis for this case included tympanosclerosis, cholesteatoma, or other middle ear masses. We review these entities and discuss their pathophysiology and implications on management.

## 1. Introduction

Tympanosclerosis is a commonly encountered physical exam finding within ENT clinics. At times, findings are not “classic” as they appear in textbooks. Knowledge of various tympanic membrane and middle ear pathologies is essential for correct diagnosis and treatment when necessary. We present a case of tympanosclerosis, which because the diagnosis was not clear on physical examination provides opportunity to review several different pathologies that may be encountered in practice.

## 2. Case Report

**Figure 1:**
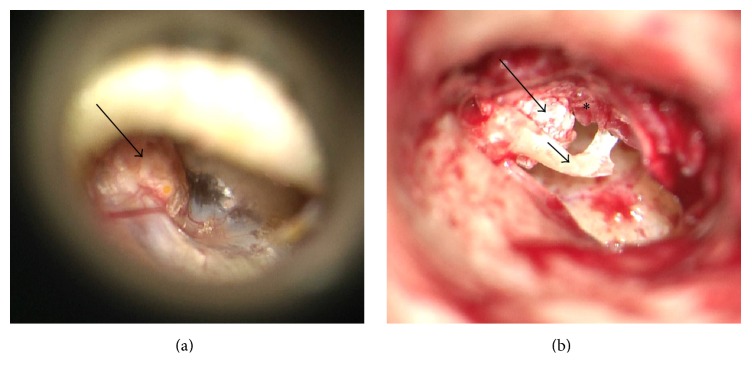
(a) View of the right tympanic membrane and lesion (arrow) utilizing the operating microscope. (b) Intraoperative view of the lesion (long arrow) within the lifted tympanic membrane (asterisk) and the underlying malleus (short arrow) (images courtesy of Dr. Abraham Jacob, MD).

An 81-year-old male presented to the ENT clinic with bilateral hearing loss, progressively worsening over the past three years. He denied otorrhea, otalgia, tinnitus, vertigo, and headaches; he had no history of ear surgery, ear trauma, or significant noise exposure. On physical exam, both auricles were normally developed, and both external auditory canals were patent with intact skin. Otoscopy revealed a diffusely thickened right tympanic membrane and a white, spherical mass involving the pars flaccida and most proximal portions of the malleus manubrium (Figure 1). Palpation revealed that the mass was firm but compressible rather than bony. The left tympanic membrane was normal, and both middle ears were aerated.

**Figure 2:**
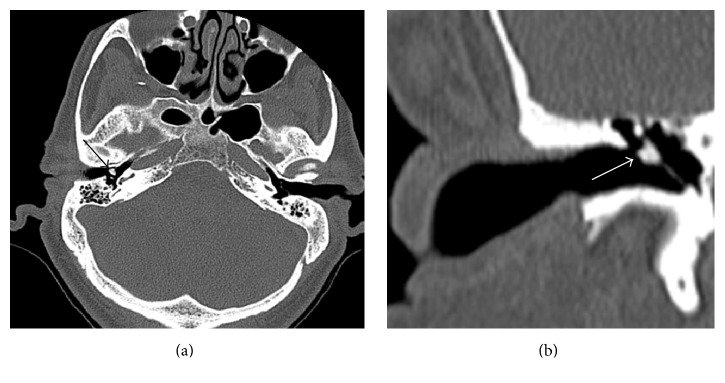
(a) Axial bone windowed temporal bone CT scan showing a 3-4 mm hyperdense lesion (arrow); the middle ear is aerated. (b) Coronal bone windowed temporal bone CT scan showing a 3-4 mm lesion lateral to the neck of the malleus (arrow). The scutum (lateral wall of epitympanum) is free of bone erosion (images courtesy of Banner University Medical Center).

**Figure 3:**
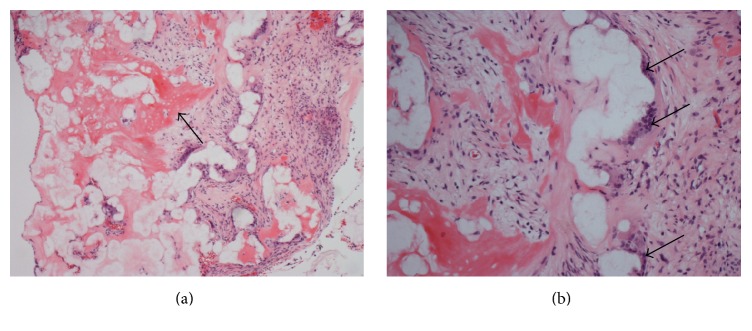
(a) Hematoxylin and eosin stained light microscopy obtained from the right ear mass (10x). Fibrous infiltrate, hyaline degeneration, and calcification (arrow) without presence of squamous epithelium are seen. (b) 20x view with black arrows demonstrating giant cell reaction (images courtesy of Sarah Tang, PSF).

An audiogram was obtained and revealed mixed hearing loss bilaterally, slightly worse on the right. A high-resolution temporal bone CT scan was then obtained and confirmed the presence of a 3-4 mm lesion involving the upper eardrum and proximal malleus (Figure 2). The middle ear and epitympanum were otherwise aerated, and despite its size and contact with the medial bony ear canal, the CT found no evidence of bone erosion. Physical examination and imaging could not provide a definitive diagnosis, and the differential diagnosis included tympanosclerosis, cholesteatoma, or less likely tumor. To establish a diagnosis, the patient was offered a right ear tympanoplasty with resection of his lesion and possible ossicular chain reconstruction (if deemed necessary intraoperatively). After discussing risks, benefits, alternatives, and reasonable expectations for informed consent, the patient elected to undergo surgery, and final pathology was consistent with tympanosclerosis, without evidence of cholesteatoma (Figure 3). The underlying ossicles were intact.

## 3. Discussion

**Figure 4:**
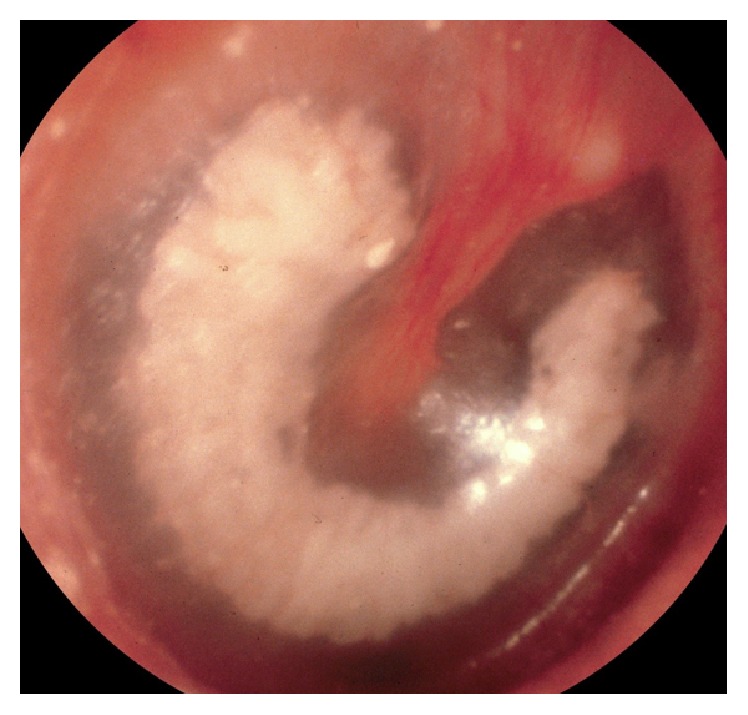
Endoscopic view of typical appearing tympanosclerosis in a right ear. Note the white plaque present within the substance of the eardrum. The middle ear is aerated (image courtesy of Dr. Abraham Jacob, MD).

Tympanosclerosis, stiffening of the tympanic membrane due to calcification, typically presents as white plaque-like lesions, involving discrete regions of the tympanic membrane and/or middle ear [1] (Figure 4). These plaques do not form spherical lesions and are typically within the drum substance itself which is normally easily discerned when using the binocular view of a microscope rather than the monocular, two-dimensional view of a hand-held otoscope. Such plaques can alter compliance of the tympanic membrane and can lead to sclerotic fixation of the ossicles [2], both of which can result in a mild conductive hearing loss. Histologically, lesions vary slightly according to their stage of development showing differing amounts of increased fibroblasts, abnormal fibers, areas of hyalinization, and areas of calcification into an osseous-like matrix [3]. Most patients require no treatment.

This patient's presentation was not typical, in that the lesion appeared to be a spherical mass rather than the plaque-like lesions seen typically in tympanosclerosis. For this reason, there was concern for cholesteatoma, a keratin-containing cyst [4], which often does appear white and spherical, as with this lesion. Cholesteatomas form via several mechanisms [5, 6] and because they expand with time and are locally destructive, surgical removal is required. They erode bone via several mechanisms, including contact under pressure, inflammation, and localized osteoclast activation [7]. To prevent recurrence, these lesions must be surgically removed in total. Given that the diagnosis was not clear, the patient was counseled regarding different possible etiologies and surgery to remove the lesion and obtain definitive diagnosis was decided upon.

Typically, a diagnosis of tympanosclerosis is easily made with the combination of history and physical exam, frequently appearing as white plaques within the substance of the tympanic membrane. When associated with hearing loss, an audiogram will reveal abnormalities involving sound conduction. In the absence of an audiogram, a tuning fork examination (512 Hz tuning fork) can be extremely helpful. The Weber exam (vibrating tuning fork placed in the midline) is lateralized to the affected ear and Rinne exam (vibrating tuning fork pressed firmly against the mastoid tip and subsequently in front of the external auditory meatus) finds bone conduction to be greater than air conduction. In this patient, the spherical appearance of this lesion prompted workup with high-resolution temporal bone CT scan (<1 mm cuts through the temporal bone) and subsequent surgery to resect and confirm the lesion histologically. Without tissue for microscopic examination, the differential for this mass included not only tympanosclerosis, but also cholesteatoma or other insidious processes such as a tumor, which made surgical excision the preferred option in this patient. Surgery was uneventful and three months after procedure the patient reported improved hearing. There was no evidence of recurrent disease on physical exam.

## Additional Points

*Presentations*. The manuscript has not been presented and is not being considered for presentation.
